# Combating Social Isolation: Evaluating the Impact of an Intergenerational Program ‘Perfect Pair’

**DOI:** 10.1093/geroni/igaf122.2778

**Published:** 2025-12-31

**Authors:** Samiya Manocha, Elizabeth Rueppel, Shriya Karmarkar, Kamryn Casey, Edie Lerner, Madison Ebstein, Celene Philip, Emily Lerner

**Affiliations:** Northwestern University Feinberg School of Medicine, Chicago, Illinois, United States; Icahn School of Medicine at Mount Sinai, New York, New York, United States; University of Michigan, Ann Arbor, Michigan, United States; University of Miami Miller School of Medicine, Miami, Florida, United States; University of Michigan-Flint, Flint, Michigan, United States; WashU Medicine, St. Louis, Missouri, United States; University of Michigan, Ann Arbor, Michigan, United States; University of Michigan, Ann Arbor, Michigan, United States

## Abstract

Social isolation and loneliness are growing public health concerns that negatively impact older and younger adults. A national report found that one in four adults aged 65+ is socially isolated, while a 2023 survey revealed that 44% of college students experience depression and 37% struggle with anxiety. Perfect Pair, a national nonprofit, combats these issues by fostering intergenerational relationships between college students and older adults. Participants are paired based on shared backgrounds and interests, meeting weekly to share stories and participate in activities like language learning and painting. A retrospective survey of participants with at least 12 meeting hours demonstrated significant benefits. Among older adult respondents (N = 39), 100% felt they gained a companion/friend, 83.3% felt less lonely, 97.2% looked forward to meetings, and 81.1% felt they have closer ties to the younger generation. Additionally, 91.9% felt increased happiness after meetings and 73% reported an improvement in their mental health. Among younger adult respondents (N = 67), 96.7% learned something new from their pair, 96.7% felt an increase in happiness after meetings, 91.8% felt they better understand aging/long-term care communities, 95.1% felt they have closer ties to the older generation, and 73.4% reported an improvement in their mental health. Perfect Pair participation led to a reduction in loneliness, increased intergenerational connection, and improved mental health for both older and younger adults. Intergenerational programming like Perfect Pair demonstrates a valuable potential to combat social isolation and loneliness through building meaningful connections.

